# Dietary Polyphenol Intake, Blood Pressure, and Hypertension: A Systematic Review and Meta-Analysis of Observational Studies

**DOI:** 10.3390/antiox8060152

**Published:** 2019-05-31

**Authors:** Justyna Godos, Marilena Vitale, Agnieszka Micek, Sumantra Ray, Daniela Martini, Daniele Del Rio, Gabriele Riccardi, Fabio Galvano, Giuseppe Grosso

**Affiliations:** 1 Department of Biomedical and Biotechnological Sciences, University of Catania, 95123 Catania, Italy; justyna.godos@student.uj.edu.pl (J.G.); fgalvano@unict.it (F.G.); 2 NNEdPro Global Centre for Nutrition and Health, St John’s Innovation Centre, Cambridge CB4 0WS, UK; s.ray@nnedpro.org.uk; 3 Department of Clinical Medicine and Surgery, “Federico II” University, 80131 Naples, Italy; marilena.vitale@yahoo.it (M.V.); riccardi@unina.it (G.R.); 4 Department of Nursing Management and Epidemiology Nursing, Faculty of Health Sciences, Jagiellonian University Medical College, 31-501 Krakow, Poland; agnieszka.micek@uj.edu.pl; 5 Wolfson College at the University of Cambridge, Cambridge CB3 9BB, UK; 6 Nutrition Innovation Centre for Food and Health at Ulster University, Coleraine BT52 1SA, UK; 7 Medical Research Council (MRC) Human Nutrition Research Unit, Cambridge CB1 9NL, UK; 8 The Laboratory of Phytochemicals in Physiology, Department of Veterinary Science, University of Parma, 43126 Parma, Italy; daniela.martini@unipr.it (D.M.); daniele.delrio@unipr.it (D.D.R.)

**Keywords:** flavonoid, anthocyanin, flavones, hypertension, blood pressure, meta-analysis, cohort

## Abstract

Background: Dietary polyphenols, including flavonoids, have been the focus of major recent attentions due to their wide content in a variety of foods commonly consumed and the findings from numerous studies showing evidence of an association with positive outcomes on human health. Methods: A systematic search using electronic databases PubMed and EMBASE was performed to retrieve English language studies published from the earliest indexing year of each database to April 2019, reporting on the association between dietary flavonoids intake and hypertension. Results: The search strategy resulted in the final selection of 20 studies including 15 cross-sectional investigations and 7 prospective cohorts (1 study reported on 3 prospective cohorts). 5 prospective cohorts, comprising 200,256 individuals and 45,732 cases of hypertension were included in the quantitative analysis. Analysis by extreme quantiles of intake of flavonoid showed a non-significant association with decreased risk of hypertension (RR (risk ratio): 0.96, 95% CI (confidence interval): 0.89, 1.03). Taking into consideration individual flavonoid subclasses, dietary anthocyanins intake was associated with 8% reduction in risk of hypertension, when comparing highest vs. lowest exposure (RR: 0.92, 95% CI: 0.88, 0.97). Conclusions: Further studies are needed to strengthen the retrieved association between anthocyanins consumption and decreased risk of hypertension and clarify whether total flavonoids or rather individual subclasses may exert beneficial effects on blood pressure.

## 1. Introduction

The reduction in cardiovascular risk and metabolic disorders associated with healthy diets have been hypothesized to be mediated, among several mechanisms, by their content in bioactive phytochemicals possibly endorsed of antioxidant and anti-inflammatory properties [1]. Dietary polyphenols have been recently put under the spotlight due to their wide content in a variety of commonly consumed foods, and to the findings from numerous studies showing significant evidence of their association with positive outcomes in the framework of human health [2,3]. Among the most studied, flavonoids represent the most heterogeneous class in terms of both chemical structure and bioactivity [4]. This class of polyphenols is characterized by C6–C3–C6 ring structure substituted with varying numbers of hydroxyl groups. The bioavailability, metabolism, and biological activity of flavonoids depends on their configuration, total number of hydroxyl groups, substitution or conjugation of functional groups, and degree of polymerization [5]. The flavonoid class is composed of several sub-classes, including anthocyanins, isoflavones, flavones, flavonols, flavanones, flavan-3-ols, and the related oligomeric proanthocyanidins. Each of these sub-classes is contained in a pattern of different foods, mostly fruits and some vegetables, but also in teas, cocoa, and some alcoholic beverages, which have been shown to exert beneficial effects toward human health [6,7,8,9,10,11,12]. Despite the now recognized inter- and intra-individual differences in absorption and metabolism, dietary flavonoids have been considered as potentially responsible, at least in part, for the beneficial effects associated to plant-based dietary patterns and of many of the benefits attributed to plant-derived beverages, such as tea and coffee [13,14]. Moreover, the dietary intake of polyphenols has been linked to higher adherence to a healthy dietary pattern [15], namely the Mediterranean diet, that has been adopted worldwide [16]. Importantly, different studies showed the association between higher adherence to Mediterranean pattern, a rich source of flavonoids, and lower prevalence of metabolic features, such as dyslipidemia [17], obesity [18,19,20,21,22], and hypertension [23,24].

There is a growing interest in the association between dietary flavonoid intake and cardio-metabolic risk factors. Results from laboratory and clinical investigations have pointed out the potential benefits of some classes of flavonoids in regulating blood pressure and endothelial function [25,26,27,28]. Studies conducted in the context of the PREDIMED (PREvencion con DIeta MEDiterranea) trial provided mechanistic evidence of association between total polyphenol excretion, blood pressure levels [29], and plasma nitric oxide production, which is a regulating factor of endothelial function [30]. However, no specific data on flavonoids was provided. Taking into account the documented heterogeneity in structural characteristics, bioavailability, absorption, and metabolism, it is of interest to summarize the existing evidence and evaluate which group of compounds provide the most robust and promising health effect. Thus, the aim of this study was to perform a meta-analysis of prospective cohort studies investigating the effects of the intake of total and individual subclasses of dietary flavonoids on the risk of hypertension.

## 2. Materials and Methods

### 2.1. Study Selection

A systematic search on electronic databases PubMed (http://www.ncbi.nlm. nih.gov/pubmed/) and EMBASE (http://www.embase.com/) was performed to retrieve English language studies published from beginning to April 2019 using the following search terms: (i) flavonoids, flavonols, flavones, anthocyanidins, flavanones, flavan-3-ols, catechins, isoflavones, proanthocyanidins, quercetin, myricetin, kaempferol; (ii) lignans, phytoestrogens; (iii) hypertension, blood pressure, cardiovascular, metabolic; and (iv) cohort, prospective, cross-sectional, longitudinal. Inclusion criteria were the following: (i) having a prospective design; (ii) evaluating the association between dietary flavonoids/lignans and/or their classes intake and risk of hypertension; (iii) assessed and reported hazard ratios (HRs) and the corresponding 95% CI (confidence interval) for hypertension (or sufficient data to compute them). No specific exclusion criteria were applied besides language restrictions. Reference lists the studies retrieved were examined to find any additional study potentially unidentified. If more than one article referred to the same cohort, only the study that included the entire cohort or with the longest follow-up was included. The selection process was independently performed by two authors (G.G. and J.G.) and retrieved articles were examined.

### 2.2. Data Extraction and Study Quality

Data were extracted from each eligible study by using a standardized extraction form. The following information was included: (1) first author name; (2) year of publication; (3) cohort name; (4) country; (5) number of participants (analyzed cohort); (6) sex of participants; (7) age range of the study population at baseline; (8) dietary food source; (9) sources used to calculate polyphenol intake; (10) follow-up period; (11) number of cases; (12) distributions of cases and person-years, HRs, and 95% CIs for all categories of exposure; (13) covariates used in adjustments. This process was independently performed by two authors (G.G. and J.G.) and discrepancies were discussed and resolved by consensus.

The quality of each study was assessed using the Newcastle-Ottawa Quality Assessment Scale [31]. The scale consists of three variables evaluating the quality of studies, including: selection (4 points), comparability (2 points), and outcome (3 points) for a total score of 9 points (9 representing the highest quality).

### 2.3. Statistical Analysis

Outcomes evaluated in the analyses included occurrence of hypertension. The analyses were performed for total flavonoid intake as well as for individual classes. HRs with 95% CI for all categories of exposure were extracted for the analysis. Random-effects models were used in order to estimate relative risks (RRs) with 95% CIs for the highest versus the lowest category of exposure. The risk estimate from the most adjusted models were used in the analysis. Heterogeneity was calculated using the Q test and *I*^2^ statistic. The level of significance for the Q test was expressed as *p* < 0.10. The *I*^2^ statistic represented the amount of total variation that could be attributed to heterogeneity. *I*^2^ values ≤25%, 25–50%, 50–75%, and >75% indicated no, small, moderate, and significant heterogeneity, respectively. A sensitivity analysis by exclusion of one study at the time was performed in order to assess the stability of results and potential sources of heterogeneity. Additional sensitivity analyses were performed to test for potential source of heterogeneity by grouping studies according to sex, geographical area, sample size, length of follow-up, and adjustment for potential confounding factors. Publication bias was evaluated by a visual investigation of funnel plots for potential asymmetry.

## 3. Results

### 3.1. Study Characteristics

**Figure 1:**
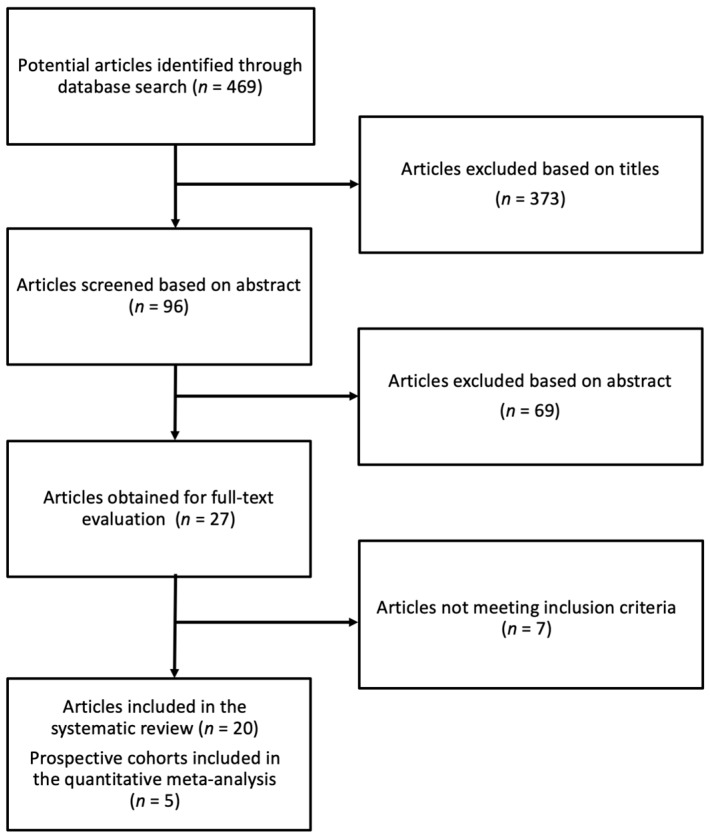
Process selection of relevant studies reporting on the association between dietary flavonoid intake and risk of hypertension.

The process of identification and study selection is summarized in Figure 1. Among the initial 469 articles screened on the basis of title, 27 articles were screened by reading full-texts. Seven studies were excluded after a full-text examination for the following reasons: two studies reported insufficient statistics, four studies reported markers of polyphenol consumption, one study provided the results only for metabolic syndrome and not hypertension or blood pressure. This search strategy resulted in the final selection of 20 studies, including 15 cross-sectional investigations and 7 prospective cohorts (1 study reported on 3 prospective cohorts). Five prospective cohorts, comprising 200,256 individuals and 45,732 cases of hypertension, were included in the quantitative analysis.

Description of main characteristics of the included studies are presented in Table 1. Cross-sectional studies included several cohorts, including the following: the Mediterranean healthy Eating, Aging, and Lifestyle (MEAL) study, the WOBASZ study, the Health Survey of Sao Paulo (ISA-Capital Study), the Framingham Offspring Study, the TOSCA.IT study, the TwinUK registry study, the Tehran Lipid and Glucose Study (TLGS), the Prospect-EPIC cohort, the Healthy Lifestyle in Europe by Nutrition in Adolescence (HELENA) study, the Korean National Health and Nutrition Examination Survey (KNHANES), the Prevención con Dieta Mediterránea (PREDIMED) study, the Alcohol and Psychosocial factors In Eastern Europe (HAPIEE) and the CARDIA study. Among prospective studies, three cohorts were conducted in the US, including the Health Professionals’ Follow-up Study (HPFS), the Nurses’ Health Study I (NHS I) and the NHS II [32]; the two cohorts conducted in Europe included the Health, the Alcohol and Psychosocial factors In Eastern Europe (HAPIEE) study [33] and the E3N, which is the French component of the European Prospective Investigation into Cancer and Nutrition (EPIC); and two conducted in Asia included Japan Public Health Center–Based Cohort II (JPHC-II) and Multi-Rural Communities Cohort (MRCohort) [34]. The cohort size varied between 301 and 87,242 individuals. Most of the studies included adult individuals ranging 40–70 years’ old. Study quality was consistently high (Table 1). A total of six studies investigated exposure to total polyphenols including various classes, six studies focused on flavonoids, two on phenolic acids, and eight studies on phytoestrogens. Among studies on flavonoid intake, TOT also reported individual subclasses, such as flavonols, flavones, flavanones, flavan-3-ols, anthocyanins, and oligomers. All studies included covariates that may have significantly influenced the endpoint outcome (hypertension), such as age, sex (when not analyzed separately), BMI, education, physical activity, and smoking status; however, not all studies adjusted for key dietary factors that might influence risk of hypertension, such as sodium and potassium intake (Table 1).

Estimation of dietary polyphenol intake was performed by using more frequently the Phenol-Explorer database, the US Department of Agriculture (USDA) Flavonoid Content of Food Database, and the European Food Information Resource (EuroFIR) Bioactive Substances in Food Information System (eBASIS) database, despite individual studies also used other databases (Table 1). The main differences across studies regarded the representativeness of the exposed cohort, as the US cohorts included individuals with demographical (i.e., post-menopausal women or adolescents), health (i.e., diabetic individuals), and social (i.e., health care workers) characteristics different from general population.

### 3.2. Total Polyphenols, Flavonoids, Phenolic Acids, and Blood Pressure/Hypertension

Total polyphenol or individual classes intake was evaluated in five studies. All studies had a cross-sectional design. In a study investigating major cardiovascular risk factors in a population of 2573 individuals (aged 50 to 75 years) with type 2 diabetes (the TOSCA.IT study), there was as significant lower mean systolic and diastolic blood pressure in the highest tertile of total polyphenol, flavonoids, and phenolic acids intake compared to the lowest one [11]. In another study conducted on 1898 women, aged from 18 to 75 y from the TwinsUK registry, intakes of total flavonoids and their subclasses (flavanones, anthocyanins, flavan-3-ols, polymers, flavonols, and flavones) were estimated from validated FFQs using an updated and extended USDA database; among the various groups of compounds tested, only anthocyanins were linearly associated with systolic blood pressure [35]. In a pan-european study conducted on adolescents participating to the HELENA study (*n* = 657) in which polyphenol intake was assessed through two 24-h recalls and evaluated using the Phenol-Explorer database, total polyphenol intake was not related with blood pressure, but sub-analyses on polyphenol classes revealed that flavonoid intake was non-significantly associated with higher diastolic blood pressure [36]. Finally, a study conducted on 8821 Polish men and women (the HAPIEE study) using a FFQ and estimation of total/individual polyphenols through the Phenol-Explorer database showed a linear inverse association between total polyphenol intake and high blood pressure (>130/85 mmHg), while, among individual classes, a significant inverse association was found with phenolic acid intake [37].

Among studies conducted outside Europe, an investigation conducted on 2618 Iranian adults, aged 19 to 84 years, showed that total polyphenol intake (assessed by a FFQ and calculated through the Phenol-Explorer database) was not related with hypertension, while among various individual classes, flavonoid intake was linearly inversely associated with systolic and diastolic blood pressure [38]. A study conducted in the context of the ‘Health Survey of Sao Paulo (ISA-Capital)’ among 550 adults and older adults in Sao Paulo, Brazil assessed total polyphenol intake through two 24-h dietary recalls reporting an inverse association between the highest tertiles of tyrosols, alkylphenols, lignans, as well as stilbenes and occurrence of hypertension; however, total polyphenol intake, and phenolic acids intake was significantly associated only in the middle tertile with hypertension, while association between flavonoids intake and hypertension was not significant [39].

Several studies have been conducted investigating individual classes or single compounds. A study from the same Brazilian cohort focused the analysis on “coffee polyphenols”, mostly including phenolic acids: the study showed a significant inverse associations between moderate intake of phenolic acids (101–337 mg/d) and hypertension [40]. Another study focusing on the relation between phenolic acids and hypertension was conducted on a sample of southern Italian adults (the MEAL cohort, *n* = 1937; age 18–92) in which polyphenol intake was assessed by a validated FFQ and calculated through the Phenol-Explorer database; individuals in the highest quartile of phenolic acid intake (median intake = 522.2 mg/day) were less likely to have hypertension, while when considering individual subclasses of phenolic acids, only hydroxyphenylacetic acids were inversely associated with hypertension [41]. Among the studies focusing the attention on one single class (or subclass) of polyphenols, an investigation conducted on 1827 men and 2918 women aged 20–69 years living in Korea (the KNHANES survey) calculated the intake of flavanols from 24-h dietary recalls using various databases on polyphenol content in foods (including the USDA Flavonoid Content of Food Database, the Japan Functional Food Factor Database, and the Rural Development Administration Food Functional Composition Table) reporting an inverse association with high blood pressure (defined as systolic blood pressure ≥130 mmHg and diastolic blood pressure ≥85 mmHg) in women but not in men [42].

### 3.3. Phytoestrogens and Blood Pressure/Hypertension

A total of eight studies investigated the association between total or individual phytoestrogen intake and blood pressure or occurrence of hypertension. A study recruiting African American and Caucasian men and women aged 18 to 30 years at baseline and following them up to 20 years; the authors reported that in multivariate adjusted models, the highest quartile of dietary isoflavone intake was associated with a 4.4 mm Hg lower SBP when compared with SBP for the lowest quartile [43]. Among studies conducted in individuals living in the Mediterranean area, participants of the MEAL cohort in the highest quartile of total phytoestrogens intake were less likely to be hypertensive, while no significant results were found for isoflavones and lignans separately; however, some individual compounds, such as biochanin A and pinoresinol showed an independent inverse association with hypertension [44]. Another study conducted in elderly individuals living in the Mediterranean area included participants of the PREDIMED trial, which showed no relation between lignan intake (estimated by Phenol-Explorer database) and blood pressure, while a significant inverse association was found when considering lignan intake together with low-fat dairy products [45]. Among studies conducted in Asian countries, where the major sources of phytoestrogens are soy products, participants of the JPHC-II (926 men and 3239 women) were examined through a FFQ including questions on soy consumption and isoflavone intake estimated with the use of Standard Tables of Food Composition in Japan, and development of high blood pressure, defined as systolic blood pressure >130 mm Hg or diastolic blood pressure >85 mm Hg or antihypertensive medication use; the study showed that isoflavones from total soy products was associated with lower risk of high blood pressure in women but not in men, while isoflavones from fermented soy product was associated in men but not in women [46]. Another study conducted on Korean men and women (*n* = 5509) aged more than 40 years old testing the association between soy isoflvones (calculated based on a database for foods consumed by Koreans and the USDA database) and high blood pressure (>130/85 mmHg) showed an inverse relation in both men and women, despite a linear association being observed only in women but not in men [47].

Given the weak estrogenic effects of phytoestrogens, some studies have been conducted focusing on post-menopausal women, which may sensibly benefit of such effect. A study conducted on 939 postmenopausal women participating in the Framingham Offspring Study aimed to assess the relation between phytoestrogen intake (assessed through self-administered FFQ and calculated by literature review) and cardiovascular risk factors, showed no significant differences between mean systolic and diastolic blood pressure across quartiles of isoflavones and lignans intake [48]. In another study conducted on 301 postmenopausal women (aged 60–75 years) living in The Netherlands exploring dietary phytoestrogen intake assessed using a FFQ covering the year prior to enrolment, no associations were found for higher intake of isoflavones and systolic and diastolic blood pressures, while it was observed lower systolic and diastolic blood pressures and a lower prevalence of hypertension associated with higher lignans intake [49]. Finally, a study conducted on post-menopausal Polish women participating to the WOBASZ and WOBASZ II surveys (*n* = 2599) assessing dietary lignans intake using a single 24-h recall and the Dutch lignan database (together with other various sources derived from the literature) reported no relation of total and individual lignans and having hypertension [50].

### 3.4. Quantitative Meta-Analysis for Dietary Flavonoid Intake and Risk of Hypertension

**Figure 2:**
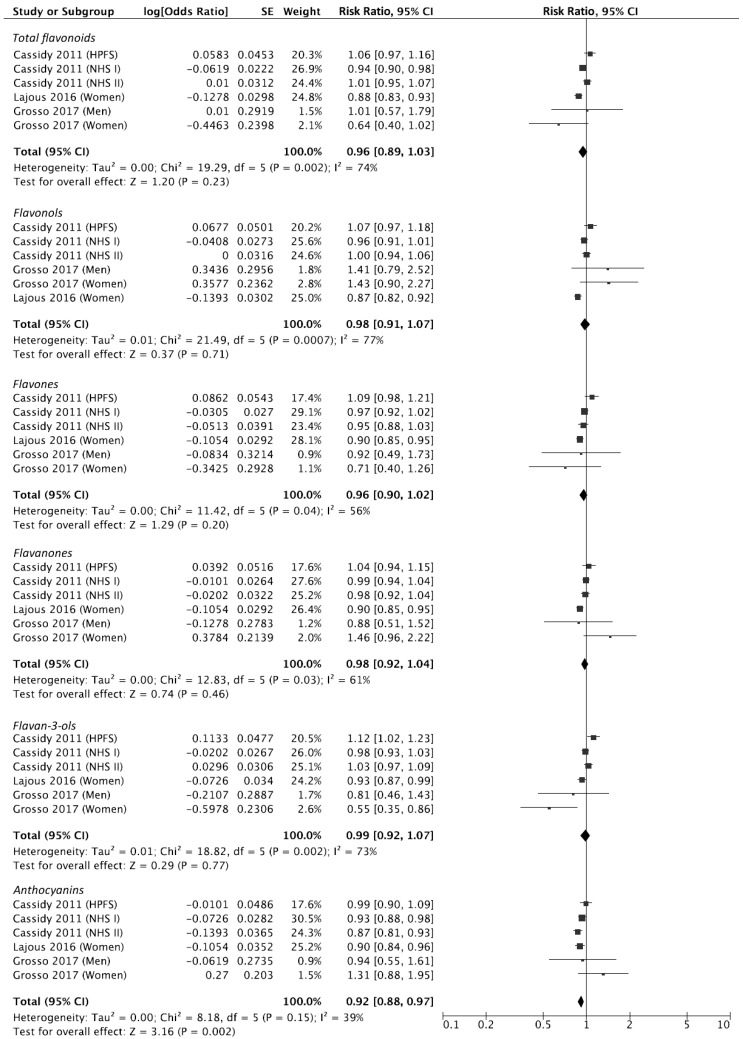
Forest plot of summary relative risks (RRs) of hypertension for the highest versus lowest (reference) category of dietary flavonoid intake.

Comparable data to perform meta-analysis of risk estimates was available only for prospective studies exploring the relation between dietary flavonoid (including all subclasses of flavonoids) intake and risk of hypertension. Forest plots comparing the highest versus the lowest intake of flavonoid intake (total and subclasses) and risk of hypertension are shown in Figure 2.

The analysis showed a non-significant association with decreased risk of hypertension (RR: 0.96, 95% CI: 0.89, 1.03; Figure 2) with significant heterogeneity (*I*^2^ = 74%, *P_heterogeneity_* = 0.01), but no evidence of publication bias (Supplementary Figure S1). Exclusion of one study at the time revealed that significant results could be retrieved when not considering the HPFS cohort, despite the exclusion not reducing heterogeneity between remaining studies. A sensitivity analysis of subgroups defined by variables that may be responsible for heterogeneity showed significant results when considering only women and studies conducted in Europe, while no further evidence was found when considering studies with larger samples and longer follow-up (Table 2).

The analysis of individual flavonoid subclasses showed significant association with hypertension risk reduction for higher intake anthocyanins with no evidence of significant heterogeneity and no evidence of publication bias at funnel plot (Supplementary Figure S1) whereas no association for extreme categories of exposure of flavonols, flavones, flavanones, and flavan-3-ols, was found (Figure 2). Sensitivity analysis conducted by excluding one study at a time revealed that also flavones were significantly associated with decreased risk of hypertension after exclusion of the HPFS cohort (with no significant heterogeneity and no evidence of publication bias) while all other results remained stably not significant.

## 4. Discussion

In this systematic review and meta-analysis, we reviewed existing studies on dietary polyphenols and blood pressure in epidemiological studies and investigated the association between total or individual subclasses of flavonoid intake and risk of hypertension in prospective cohort studies. The epidemiological studies evaluating the association between total dietary polyphenols and cardio-metabolic risk factors, including hypertension, generally showed contrasting results. Among articles with cross-sectional design, while no study but one [38] reported an inverse association between total polyphenol or flavonoid intake and occurrence of hypertension, few studies reported significant results for phenolic acids [40,41]. The findings on the relation between dietary phytoestrogens intake and blood pressure are more univocal, with general significant inverse association between intake of either isoflavones or lignans and blood pressure levels, despite heterogeneity of results between sexes and the cross-sectional design of most of the studies reviewed limiting the level of evidence. Among subclasses of flavonoids, the meta-analysis showed that dietary intake of anthocyanins was significantly associated with reduced risk of hypertension: comparison of the highest vs. lowest anthocyanins exposure showed an 8% reduction in risk of hypertension. Moreover, after exclusion of data from one cohort, also the analyses for total flavonoids and flavones demonstrated significant results. Significant heterogeneity between studies observed for total flavonoids should be considered as a major limitation of these findings. Nevertheless, heterogeneity was mainly related to the strength of the association rather than to the direction of risk estimates, suggesting overall promising perspectives for future studies.

Existing literature on studies aiming to explore the relation between flavonoid-rich foods, dietary flavonoids and hypertension is generally consistent but not univocal. Two meta-analyses of dietary interventions characterized by isocaloric diets with pulses or fruits and vegetables (including one portion of berries/day and 50 g of dark chocolate) showed significant reduction in systolic/diastolic blood pressure [51] and of markers of endothelial function [52], respectively. Among major food sources of anthocyanins, potential blood pressure lowering effects have been demonstrated for pomegranate juice [53] and berries [54,55], whereas a lack of effects has been reported for blueberry supplementation [56]. Among food sources of isoflavones, a meta-analysis exploring the effects of soybeans on blood pressure in postmenopausal women showed that ingestion of ≥25 g soy protein per day had blood pressure-lowering effects, and the improvements in blood pressure may be due to the isoflavones component of soy protein [57]. Other sources of phytoestrogens are seeds, which are rich in lignans: meta-analyses on clinical trials evaluating the effects of flaxseeds and sesame consumption on blood pressure concluded that they both may exert blood pressure-lowering effects [58,59]. Among the major sources of phenolic acids, the most comprehensive meta-analysis of cohort studies on coffee consumption and incidence of new-onset hypertension reported a modest decrease in risk of hypertension [60], while several meta-analyses investigating the effects of tea on blood pressure, showing potential benefits for long-term (>12 weeks) intake [61,62] and significant effects also for secondary prevention of cardiovascular disease among overweight and obese adults [63] and in individuals within pre-hypertensive and hypertensive ranges [64]. However, meta-analysis of clinical trials on polyphenol supplementation showed contrasting results. A previous meta-analysis of randomized controlled trials on the effects of anthocyanins on blood pressure showed overall null results [65]. Other meta-analyses on soy isoflavones supplementation reported that soy isoflavone extracts significantly decreased systolic but not diastolic blood pressure in adult humans, and no dose–response relationship was observed [66], while another meta-analysis suggested that the effects were more evident in hypertensive, but not in normotensive individuals [67]. Similarly, the beneficial potential of flaxseed to reduce blood pressure may be greater when it is consumed as a whole seed rather than as lignan extract [68]. Possible reasons for such scarce findings may depend on limitations in the study design of the trials included in the analysis, or on the short-term consumption to which the trials refer, which may be indicative of potential acute effects, but not equivalent to long term consumption. Moreover, supplementation using pharmaceutical formulas (i.e., pills or capsules) of pure extracts may not be equivalent to dietary intake. Finally, it has been suggested that flavonoid bioactivity may not follow a classical linear dose–response association, and attention should be payed when designing and pooling together results from randomized controlled trials [69].

Flavonoids have been hypothesized to exert beneficial effects towards the cardiovascular system through their antioxidant and antiradical action, exerted through a variety of molecular mechanisms [70], and importantly, as demonstrated in both experimental and clinical studies, altered oxidative imbalance is linked to higher cardiovascular risk [71,72,73,74]. Furthermore, flavonoids may also act more specifically as signaling molecules, interacting with cellular receptors or proteins that are involved in regulatory pathways, resulting in physiological responses or regulation of gene expression [70]. Regarding their potential effects on endothelial function and regulation of vasodilatory processes associated with blood-flow, flavonoids can act as inhibitors of endothelial NAD(P)H oxidase, which has been linked to the regulation of nitric oxide (NO) levels in the vascular endothelium via inhibition of superoxide production [75]. Specific classes of phenolic compounds or their metabolites have been reported to improve endothelial dysfunction through their antioxidant attitude, but also directly acting over NO metabolism or reducing vasoconstriction by acting on ACE and angiotensin II receptor activity [76]. In particular, isoflavones have demonstrated interaction with the estrogen-response element of genes related to endothelial NO synthase leading to increase in endogenous NO production [77]. Furthermore, in several studies, flavonoids were shown to modulate inflammation through inflammatory cell infiltration, lowered levels of pro-inflammatory cytokines, and improved cell survival and function. These anti-inflammatory effects were mediated via inhibition of NF-κB [78,79]. Concerning anthocyanins, the only subclass of flavonoids that was significantly associated with decreased risk of hypertension in the present study, their effects involve targeting the NF-κB signaling pathway, activator protein 1(AP-1), the Phase II enzyme activation and Nrf2, and the mitogen-activated protein kinase (MAPK) signaling pathway [80]. Anthocyanins were shown to exert inhibitory action against vasodilators. In example, blackberry juice containing cyanidin-3-*O*-glucoside was shown to exert protective effects against endothelial dysfunction and vascular failure via scavenging of peroxynitrate, a strong oxidant responsible for DNA and protein damage [81]. In line with those results, another study demonstrated that anthocyanin fraction of blackberry extract was able to suppress NO production via inhibition of inducible NO synthase due to the attenuation of NK-κB and MAPK signaling pathways [82]. Cyanidin-3-*O*-glucoside was shown to regulate the phosphorylation of endothelial NO synthase, thus ameliorating endothelial dysfunction and regulating blood pressure [83,84]. Finally, delphinidin, an anthocyanin present in red wine, was demonstrated to inhibit endothelial cell apoptosis via NO pathway and regulation of calcium homeostasis [85]. However, when taking into account the results obtained for anthocyanins, it should be considered that anthocyanins are relatively unstable molecules, as they undergo re-arrangements in response to pH. Depending on the nature of compound and sugar conjugation, anthocyanins were shown to reach the human colon after ingestion [86]. Despite laboratory studies providing the biological rationale for the potential association of dietary flavonoids and, in particular, anthocyanins and risk of hypertension, the overall results need to be further clarified by additional experimental and epidemiological studies.

Some limitations of the included studies may explain the retrieved heterogeneity of results and the lack of significance of certain analyses. First, differences in polyphenol food content databases used among studies may lead to different ‘weighting’ of food items selected in terms of flavonoid content. Moreover, only some compounds are known to exert beneficial effects toward the endothelium [87], and intra- and inter-individual differences in absorption and metabolism of such compounds may enhance or reduce such activity. Secondly, food items included in dietary questionnaires comprised different numbers of foods containing flavonoids, thus further potentially biasing the real content of polyphenols. Additionally, studies with only baseline evaluation of food intake may not have taken into account possible changes of the diet over the follow-up period. However, as the exposure was ascertained before the diagnosis of hypertension, misclassification would tend to underestimate the true associations. Moreover, the role of individual dietary compounds is difficult to disentangle, as intake of polyphenols has been reported to correlate with the intake of fruits and vegetables and their constituents—i.e., vitamin C, vitamin E, carotenoids, folate, and fiber—which may confound or act synergistically toward the risk of chronic diseases [88]. Finally, subgroup analyses showed significant results only among women. A possible explanation for this is the natural differences occurring between men and women due to hormonal protection in the latter before menopause. However, these potential differences need to be further investigated.

## 5. Conclusions

In conclusion, results from this study are promising but not conclusive. Further studies are needed to elucidate the retrieved association between polyphenol consumption and decreased risk of hypertension and to clarify whether individual subclasses, rather than the total content of polyphenols, may exert beneficial effects on blood pressure.

## Figures and Tables

**Table 1:** Characteristics of prospective cohort studies investigating the relation between dietary polyphenol intake and blood pressure/hypertension

Author, Year	Cohort Name, Country	Study Design	Follow-Up (Years of Study)	Total Population (Analyzed Sample)	Cases (Hypertension/High Blood Pressure)	Sex	Age	Dietary Assessment	Polyphenol Estimation	Polyphenol Classes	Hypertension Assessment	Adjustments
Vitale, 2006 [11]	TOSCA.IT, Italy	Cross-sectional	NA	2573	NR	MF	50–75 y (range)	248-item FFQ	USDA and Phenol-Explorer database	Flavonoids, phenolic acids	Blood pressure measured during clinical visit	Gender, age, BMI, waist and hip circumferences, smoke, alcohol intake, dietary lipids, SFA, MUFA, PUFA, added sugars, and fiber.
Cassidy, 2011 [32]	NHS I, USA	Prospective	14 y (1990–2004)	46,672	17,616	F	30–55 y (range)	FFQ	USDA and EuroFIR database, published literature	Flavonoids (and subclasses)	Self-reported	Age and smoking, BMI, PA, alcohol consumption, family history of hypertension, aspirin use, multivitamin use, and intakes of sodium, magnesium, potassium, fiber, whole grain, folate, and caffeine.
Cassidy, 2011 [32]	NHS II, USA	Prospective	14 y (1991–2005)	87,242	11,402	F	25–42 y (range)	FFQ	USDA and EuroFIR database, published literature	Flavonoids (and subclasses)	Self-reported	Age and smoking, BMI, PA, alcohol consumption, family history of hypertension, aspirin use, multivitamin use, and intakes of sodium, magnesium, potassium, fiber, whole grain, folate, and caffeine.
Cassidy, 2011 [32]	HPFS, USA	Prospective	14 y (1990–2004)	23,043	5,629	M	40–75 y (range)	FFQ	USDA and EuroFIR database, published literature	Flavonoids (and subclasses)	Self-reported	Age and smoking, BMI, PA, alcohol consumption, family history of hypertension, aspirin use, multivitamin use, and intakes of sodium, magnesium, potassium, fiber, whole grain, folate, and caffeine.
Grosso, 2017 [33]	HAPIEE, Poland	Prospective	4 y (2002–2005)	2725	1735	MF	45–69 y (range)	148-item FFQ	Phenol-Explorer database	Total polyphenols, flavonoids (and subclasses), phenolic acids (and subclasses), stilbenes, lignans, others	Blood pressure measured during clinical visit	Age, total energy intake, BMI, PA, educational status, smoking status, alcohol consumption sodium, potassium and fiber intake, menopausal status (women only).
Lajous, 2016 [34]	E3N, France	Prospective	14 y (1993–2008)	40,574	9350	F	45–58 y (range)	208-item FFQ	Phenol-Explorer database	Flavonoids (and subclasses)	Self-reported	Education, family history of hypertension, diabetes, hypercholesterolemia, BMI, smoking, PA metabolic equivalents per week, hormone therapy, alcohol, processed meat, caffeine, magnesium, potassium, and omega-3.
Jennings, 2012 [35]	TwinsUK, UK	Cross-sectional	NA	1898	NR	F	18–75 y (range)	131-item FFQ	USDA and Phenol-Explorer database	Flavonoids (and subclasses)	Blood pressure measured during clinical visit	Age, current smoking, physical activity, BMI, use of hormone replacement therapy, use of blood pressure or statin medication, use of vitamin supplements, use of oral contraceptives, menopausal status, family history of heart disease or hypertension, and intakes of energy, alcohol, SFAs, MUFAs, PUFAs, fiber, and sodium.
Wisnuwardani, 2019 [36]	HELENA, multicenter Europe	Cross-sectional	NA	657	NR	MF	12.5–17.5 y (range)	24 h dietary recalls	Phenol-Explorer database	Total polyphenols	Blood pressure measured during clinical visit	Age, sex, European region, education of mother, education of father, puberty status, BMI, monosaccharides and disaccharides, polysaccharides, fibre, monounsaturated fatty acids, saturated fatty acids, cholesterol, protein, vitamin C, and energy intake.
Grosso, 2017 [37]	HAPIEE, Poland	Cross-sectional	NA	8821	5.341	MF	45–69 y (range)	148-item FFQ	Phenol-Explorer database	Total polyphenols, flavonoids, phenolic acids, stilbenes, lignans, others	Blood pressure measured during clinical visit	Age, gender, education, physical activity, smoking status, alcohol drinking, BMI, and total energy intake.
Sohrab, 2013 [38]	TLGS, Iran	Cross-sectional	NA	2618	NR	MF	19–84 y (range)	168-item FFQ	USDA and Phenol-Explorer database	Total polyphenols, flavonoids, phenolic acids, stilbenes, lignans	Blood pressure measured during clinical visit	Age, gender, physical activity, smoking status and educational levels, study center, and total energy intake and BMI.
Miranda, 2016 [39]	ISA-Capital Study, Brazil	Cross-sectional	NA	550	267	MF	≥12 y	24 h dietary recalls	Phenol-Explorer database	Total polyphenols, flavonoids, phenolic acids, stilbenes, lignans, others (and subclasses)	Blood pressure measured during home visit	Age, sex, race, educational level, BMI, smoking, physical activity, intake of sodium, fiber, saturated fat, alcohol, total energy intake, misreporting, and other polyphenol components.
Miranda, 2017 [40]	ISA-Capital Study, Brazil	Cross-sectional	NA	557	NR	MF	45.1 y (mean)	24 h dietary recalls	Phenol-Explorer database	Coffee polyphenols (phenolic acids and others)	Blood pressure measured during home visit	Age, sex, race, BMI, smoking, alcohol, physical activity, household per capita income, intake of caffeine, added sugars, total energy intake, and saturated fat, other polyphenol intake.
Godos, 2017 [41]	MEAL, Italy	Cross-sectional	NA	1936	976	MF	≥18 y	110-item FFQ	Phenol-Explorer database	Phenolic acids (and subclasses and individual polyphenols)	Blood pressure measured during clinical visit	Age, energy intake, BMI, smoking status, alcohol consumption, physical activity level, educational level, occupational level, menopausal status, fiber, sodium, potassium, magnesium, and calcium intake, adherence to the Mediterranean diet.
Yang, 2012 [42]	KNHANES, Korea	Cross-sectional	NA	4745	NR	MF	20–69 y (range)	24 h dietary recalls	USDA database, Japan Functional Food Factor Database, Rural Development Administration Food Functional Composition Table	Flavanols	Blood pressure measured during clinical visit	Age, BMI, education, current drinking, vitamin/mineral supplement use, functional food use, intakes of total energy, fat, fiber, and carbohydrate.
Richardson, 2016 [43]	CARDIA, USA	Cross-sectional	NA	3142	843	MF	18–30 y (range)	100-item FFQ	USDA database	Isoflavones	Blood pressure measured during clinical visit	Age, sex, BMI, smoking, alcohol intake, physical activity, total energy, and sodium intake.
Godos, 2018 [44]	MEAL, Italy	Cross-sectional	NA	1936	976	MF	≥18 y	110-item FFQ	Phenol-Explorer database	Total phytoestrogens, isoflavones (and individual polyphenols), lignans (and individual polyphenols)	Blood pressure measured during clinical visit	Age, energy intake, smoking status, alcohol consumption, physical activity level, educational level, occupational level, sodium and potassium intake.
Creus-Cuadros, 2017 [45]	PREDIMED, Spain	Cross-sectional	NA	7169	NR	MF	~67 y (mean)	137-item FFQ	Phenol-Explorer database	Lignans	Blood pressure measured during clinical visit	Recruitment center, sex, age, smoking, soft drinks, carbohydrates, saturated fatty acids, monounsaturated fatty acids, polyunsaturated fatty acids, n3 fatty acids, and dairies.
Nozue, 2017 [46]	JPHC II, Japan	Prospective	5 y (1993–NR)	4165	1335	MF	40–69 y (range)	52-item FFQ	Published literature	Isoflavones	Blood pressure measured during clinical visit	Blood pressure at baseline, age, BMI, alcohol consumption, smoking status, physical activity, energy, energy-adjusted salt, saturated fats, green and yellow vegetables, pickled vegetables, fruits and fish, public health status, and menopausal status.
Woo, 2018 [47]	MRCohort, South Korea	Prospective	3.6 y (median, 2005–2013)	5509	NR	MF	≥40 y	106-item FFQ	USDA database	Isoflavones	Blood pressure measured during clinical visit	Age, calcium intake, fiber intake, egg consumption in both men and women, soda drink consumption for isoflavones analysis in men and current smoking, higher education, regular exercise, and total energy intake for soy protein/isoflavones analyses in women.
de Kleijn, 2002 [48]	Framingham Offspring Study, USA	Cross-sectional	NA	939	NR	F	59 ± 7.5 y (mean)	130-item FFQ	Published literature	Isoflavones, lignans	Blood pressure measured during clinical visit	Age, BMI, use of hormone replacement therapy, smoking, dietary fiber intake, and potassium intake.
Kreijkamp-Kaspers, 2004 [49]	Prospect-EPIC, The Netherlands	Cross-sectional	NA	301	113	F	60–75 y (range)	FFQ	Published literature	Isoflavones, lignans	Blood pressure measured during clinical visit	Age, cholesterol lowering medication, use of antihypertensives, BMI, waist/hip ratio, smoking, physical activity, total energy intake, alcohol intake, saturated fat, mono-unsaturated fat, polyunsaturated fat, time postmenopausal, fiber intake, fruit intake, vegetable intake, and vitamin C intake.
Witkowska, 2018 [50]	WOBASZ and WOBASZ II, Poland	Cross-sectional	NA	2599	1635	F	≥20 y	24 h dietary recalls	Published literature	Lignans (and individual polyphenols)	Blood pressure measured during clinical visit	Age, smoking, BMI, alcohol intake, education, leisure-time physical activity, glucose level, cholesterol level, menopause hormone therapy, energy intake.

Abbreviations: CARDIA (Coronary Artery Risk Development in Young Adults); E3N (Etude Epidémiologique auprès des femmes de la Mutuelle Générale de l’Education Nationale); EPIC (European Prospective Study into Nutrition and Cancer); F (Female); FFQ (Food Frequency Questionnaire); HAPIEE (Health, Alcohol, and Psychosocial factors In Eastern Europe); HELENA (Healthy Lifestyle in Europe by Nutrition in Adolescence); HPFS (Health Professionals Follow-Up Study); JPHC II (Japan Public Health Center–Based Cohort II); KNHANES (Korean National Health and Nutrition Examination Survey); M (Male); MEAL (Mediterranean healthy Eating, Ageing, and Lifestyle); MRCohort (Multi-Rural Communities Cohort); NA (Not applicable); NHS I (Nurses’ Health Study I); NHS II (Nurses’ Health Study II); NR (Not Reported); PA (physical activity); PREDIMED (Prevención con Dieta Mediterránea); TGLS (Tehran Lipid and Glucose Study); USDA (US Department of Agriculture).

**Table 2:** Subgroup analyses of studies reporting risk of hypertension for the highest versus lowest (reference) category of dietary flavonoids

Subgroup/Additional Analysis	No. of Cohorts	RR (95% CI)	*I*^2^	*P_heterogeneity_*
Total	5	0.96 (0.89, 1.03)	74%	0.002
Sex				
Male	2	1.06 (0.97, 1.16)	0%	0.87
Female	4	0.93 (0.87, 1.00)	76%	0.005
Geographical area				
Europe	2	0.88 (0.83, 0.93)	0%	0.37
North America	3	0.99 (0.93, 1.07)	73%	0.02
Number of cases				
>10,000	2	0.97 (0.91, 1.04)	72%	0.06
<10,000	3	0.93 (0.79, 1.10)	79%	0.003
Follow-up				
>10y	4	0.97 (0.90, 1.04)	82%	0.0009
<10y	1	0.78 (0.50, 1.21)	31%	0.23
Adjusted for smoking				
No	0	NA	NA	NA
Yes	5	0.96 (0.89, 1.03)	74%	0.002
Adjusted for potassium/sodium				
No	0	NA	NA	NA
Yes	5	0.96 (0.89, 1.03)	74%	0.002
Adjusted for physical activity				
No	0	NA	NA	NA
Yes	5	0.96 (0.89, 1.03)	74%	0.002
Adjusted for BMI				
No	0	NA	NA	NA
Yes	5	0.96 (0.89, 1.03)	74%	0.002
Adjusted for education				
No	3	0.99 (0.93, 1.07)	73%	0.02
Yes	2	0.88 (0.83, 0.93)	0%	0.37

## Supplementary Materials

The following are available online at https://www.mdpi.com/2076-3921/8/6/152/s1, Figure S1: Funnel plot for hypertension risk of the highest versus lowest (reference) category of dietary flavonoid intake, Table S1: PRISMA checklist.
